# Serotoninergic receptor ligands improve Tamoxifen effectiveness on breast cancer cells

**DOI:** 10.1186/s12885-021-09147-y

**Published:** 2022-02-15

**Authors:** Maria Rosaria Ambrosio, Elisa Magli, Giuseppe Caliendo, Rosa Sparaco, Paola Massarelli, Vittoria D’Esposito, Teresa Migliaccio, Giusy Mosca, Ferdinando Fiorino, Pietro Formisano

**Affiliations:** 1 Institute for Experimental Endocrinology and Oncology “G. Salvatore” – National Research Council (IEOS-CNR), Via Pansini 5, 80131 Naples, Italy; 2 Department of Translational Medicine, University of Naples “Federico II” (DiSMeT-UniNa), Via Pansini 5, 80131 Naples, Italy; 3 Department of Pharmacy, University of Naples “Federico II” (UniNa), Via Montesano 49 -, 80131 Naples, Italy; 4 Department of Medicine, Surgery and Neuroscience, University of Siena, Strada delle Scotte 6 –, 53100 Siena, Italy

**Keywords:** Serotonin, Breast cancer, Serotoninergic receptor ligands, Tamoxifen resistance, Connective Tissue Growth Factor

## Abstract

**Background:**

Serotonin (or 5-Hydroxytryptamine, 5-HT) signals in mammary gland becomes dysregulated in cancer, also contributing to proliferation, metastasis, and angiogenesis. Thus, the discovery of novel compounds targeting serotonin signaling may contribute to tailor new therapeutic strategies usable in combination with endocrine therapies. We have previously synthesized serotoninergic receptor ligands (SER) with high affinity and selectivity towards 5-HT_2A_ and 5-HT_2C_ receptors, the main mediators of mitogenic effect of serotonin in breast cancer (BC). Here, we investigated the effect of 10 SER on viability of MCF7, SKBR3 and MDA-MB231 BC cells and focused on their potential ability to affect Tamoxifen responsiveness in ER^+^ cells.

**Methods:**

Cell viability has been assessed by sulforhodamine B assay. Cell cycle has been analyzed by flow cytometry. Gene expression of 5-HT receptors and Connective Tissue Growth Factor (CTGF) has been checked by RT-PCR; mRNA levels of CTGF and ABC transporters have been further measured by qPCR. Protein levels of 5-HT_2C_ receptors have been analyzed by Western blot. All data were statistically analyzed using GraphPad Prism 7.

**Results:**

We found that treatment with SER for 72 h reduced viability of BC cells. SER were more effective on MCF7 ER^+^ cells (IC_50_ range 10.2 μM - 99.2 μM) compared to SKBR3 (IC_50_ range 43.3 μM - 260 μM) and MDA-MB231 BC cells (IC_50_ range 91.3 μM - 306 μM). This was paralleled by accumulation of cells in G0/G1 phase of cell cycle. Next, we provided evidence that two ligands, SER79 and SER68, improved the effectiveness of Tamoxifen treatment in MCF7 cells and modulated the expression of CTGF*,* without affecting viability of MCF10A non-cancer breast epithelial cells. In a cell model of Tamoxifen resistance, SER68 also restored drug effect independently of CTGF.

**Conclusions:**

These results identified serotoninergic receptor ligands potentially usable in combination with Tamoxifen to improve its effectiveness on ER^+^ BC patients.

**Supplementary Information:**

The online version contains supplementary material available at 10.1186/s12885-021-09147-y.

## Background

Serotonin (5-HT) is a biogenic amine acting as neurotransmitter in the nervous system both at central and peripheral level [1–4]. Besides playing a role in several physiological and pathological processes, including circadian rhythms, sexual and feeding behavior, thermoregulation and cardiovascular function [5–9], 5-HT acts as trophic, mitogenic and anti-apoptotic factor for a wide range of normal and tumor cells [10–13]. Indeed, a growth stimulatory effect of 5-HT on prostate, small-cell lung, colorectal, hepatocellular and breast carcinoma, cholangiocarcinoma, glioma, bladder cancer and ovarian tumors has been described [14]. 5-HT also promotes cancer cell migration, invasiveness and angiogenesis [15]. The multiple, sometimes opposing, actions of serotonin occur through the interaction with a wide range of receptors. Indeed, with the exception of 5-HT_3_, the unique receptor involving an ion channel that regulates the flow of sodium and potassium ions, six classes of 5-HT receptors - including additional subclasses - named 5-HT_1_, 5-HT_2_, 5-HT_4_, 5-HT_5_, 5-HT_6_ and 5-HT_7_, are G-protein-coupled [16]. More often the mitogenic effect of 5-HT is mediated by 5-HT_1_ and 5-HT_2_ receptors while less frequently through 5-HT_4_ and 5-HT_6_ [15]. Serotonin plays a central role in mammary gland ensuring epithelial homeostasis during changes associated with pregnancy, lactation and involution [17]. Thus, an extensive alteration of 5-HT signaling may contribute to breast cancer (BC) phenotype [15]. Of note, BC cells produce and secrete high levels of serotonin that, interfering with mitochondria biogenesis, confers proliferative advantages [18]. BC is the most common cancer in women worldwide [19], with estrogen receptor positive (ER^+^) BC representing approximately 75% of all diagnosed cancers [20]. About the latter, the predominant treatment strategy consists in the inhibition of ER pathway at various levels, including the use of selective estrogen receptor modulators (SERMs), like Tamoxifen, to directly antagonize the receptor [21]. However, Tamoxifen effectiveness may be modulated by the interaction with several drugs, including those acting on serotonin signaling. Nevertheless, serotonin action has also been targeted by using 5-HT antagonists and/or uptake inhibitors to prevent cancer cell growth [15]. Our research group has been involved in the synthesis of serotoninergic receptor ligands (SER) with high affinity and selectivity [22–25]. Here, we analyzed a set of previously synthetized SER with affinity and selectivity binding profile towards 5-HT_2A_ and 5-HT_2C_ receptors, known as mediators of mitogenic effect of serotonin in BC cells [18, 26]. We found that some of these serotoninergic receptor ligands improve Tamoxifen responsiveness in MCF7 BC cells and that such effect occurs through the modulation of CTGF (Connective Tissue Growth Factor) expression. Overall, these results suggest these compounds as new serotoninergic receptor ligands potentially useful to ameliorate Tamoxifen effectiveness in ER^+^ BC cells.

## Methods

### Materials

Media, sera and antibiotics for cell culture were from Lonza (Basel, Switzerland). Reagents and substituted piperazines for synthesis of SER, Estradiol and Tamoxifen for cell treatments and all other chemicals were from Sigma-Aldrich (St Louis, MO, USA). TRIzol solution for RNA isolation, SuperScript III Reverse Transcriptase with oligo dT primers for RNA reverse transcription and AmpliTaq Gold for RT-PCR were from Life Technologies (Carlsbad, CA, USA). iTaq Universal SYBR Green Supermix for Quantitative Real-Time PCR (qPCR) was from Biorad (Hercules, CA, USA). 5HT_2C_ and Vinculin antibodies for Western Blot were from Santa Cruz (Dallas, TX, USA). Secondary antibody (Anti-mouse 1:2000) was purchased from Bio-Rad (Hercules, CA, USA).

### Synthesis of serotoninergic receptor ligands and in vitro receptor binding

All reactions were monitored by TLC, carried out on Merck 60G F_254_ plates with fluorescent indicator and the plates were visualized with UV light (254 nm). Each final compound and intermediate was purified by silica gel column chromatography (Macherey-Nagel 60 0,063–0,2 mm/70–230 mesh). Some final compounds were obtained in a pure form after conversion in the corresponding hydrochloride salts. ^1^H-NMR and ^13^C-NMR spectra were recorded on Varian Mercury Plus 400 MHz instrument. Unless otherwise stated, all spectra were recorded in CDCl_3_. Chemical shifts are reported in ppm using Me_4_Si as internal standard. The following abbreviations are used to describe peak patterns when appropriate: s (singlet), d (doublet), t (triplet), m (multiplet), q (quartet), qt (quintet), dd (double doublet), ddd (double dd), bs (broad singlet). Mass spectra of the final products were performed on LTQ Orbitrap XL™ Fourier transform mass spectrometer (FTMS) equipped with ESI ION MAX™ source (Thermo Fisher, San José, USA). Melting points were determined using a Buchi B-540 hot-stage instrument and are uncorrected. Where analyses are indicated only by the symbols of the elements, results obtained are within ±0.4% of the theoretical values. Solutions were dried over Na_2_SO_4_ and concentrated with Buchi R-114 rotavapor at low pressure. Once synthesized, SER were tested for in vitro affinity for serotonin 5-HT_1A_, 5-HT_2A_ and 5-HT_2C_ receptors by radioligand binding assays. The more active compounds on serotonin receptors have been selected and evaluated for their affinity for dopaminergic (D_1_ and D_2_) and adrenergic (α_1_ and α_2_) receptors. All the compounds were dissolved in 5% DMSO. The following specific radioligands and tissue sources were used: (a)serotonin 5-HT_1A_ receptor, [^3^H]-8-OH-DPAT, rat brain cortex; (b) serotonin 5-HT_2A_ receptor, [^3^H]ketanserin, rat brain cortex; (c)serotonin 5-HT_2C_ receptor, [^3^H]mesulergine, rat brain cortex. Non-specific binding was determined as described in the experimental section, and specific binding as the difference between total and non-specific binding. Blank experiments were carried out to determine the effect of 5% DMSO on the binding and no effects were observed. Competition experiments were analyzed by PRISM 5 (GraphPadPrism®, 1992–2007, GraphPad Software, Inc., La Jolla, CA, USA) to obtain the concentration of unlabeled drug that caused 50% inhibition of ligand binding (IC_50_), with six concentrations of test compounds, each performed in triplicate. The IC_50_ values obtained were used to calculate apparent inhibition constants (K_i_) by the method of Cheng and Prussoff [27], from the following equation: K_i_ = IC_50_/(1 + S/K_D_) where S represents the concentration of the hot ligand used and K_D_ its receptor dissociation constant (K_D_ values, obtained by Scatchard analysis [28], were calculated for each labeled ligand). Radioligand binding assays for 5-HT_1A_ were performed following a published procedure [29]. 5-HT_2A_ and 5-HT_2C_ binding assays were performed reported by Herndon et al. [30].

### Cell cultures

MCF7 (ER^+^, PR^+^, HER2^−^), SKBR3 (ER^−^, PR^+^, HER2^+^) and MDA-MB231 (ER^−^, PR^−^, HER2^−^) human BC cells and MCF10A non-cancer breast epithelial cells were available in our laboratory. MCF7, SKBR3 and MDA-MB231 cells were cultured in DMEM, supplemented with 10% FBS, 2 mM glutamine, 100 units/ml penicillin and 100 units/ml streptomycin. MCF10A cells were cultured in MEBM, supplemented with 0.4% BPE, 0.1% hEGF, 0.1%, Insulin, 0.1% Hydrocortisone and 0.1% GA-1000. Cultures were maintained in a humidified atmosphere of 95% air and 5% CO2 at 37 °C. Treatment with SER were carried out in culture conditions. Treatment with Tamoxifen and/or SER were carried out upon 48 h estrogen starvation in phenol-red free medium supplemented with 10% Charcoal Stripped (C/S) FBS, 2 mM glutamine, 100 units/ml penicillin and 100 units/ml streptomycin.

### Cell survival assay

Cells were fixed with 50% trichloroacetic acid for at least 2 h at 4 °C, washed with distilled and de-ionized water, air-dryed and stained 30 min with 0.4% sulforhodamine B in 1% acetic acid. Unbound dye was removed and 10 mM tris-HCl solution (pH 7.5) was added to dissolve the protein-bound dye. Cell survival was assessed by optical density determination at 510 nm using a microplate reader [31].

### Establishing of Tamoxifen-resistant model (MCF7-R)

MCF7 cells were cultured for 4 months in phenol-red free medium supplemented with 10% (C/S) FBS and continuously exposed to Tamoxifen (1 μM). At the end, the acquisition of drug resistance was measured treating the cells with increasing concentration of Tamoxifen (100 nM to 6 μM) for 72 h before measuring cell survival by sulforhodamine B assay. To further validate the degree of drug resistance, the expression levels of ABCC1, ABCG1 and ABCG2 – members of ABC transporter family known as involved in multi-drug resistance – were evaluated by Quantitative Real-Time PCR (qPCR; see below) upon cell treatment with 5 μM Tamoxifen for 72 h.

### Cytofluorimetric analysis

Cells were collected and fixed in 70% (v/v) ethanol for at least 2 h at −20 °C. Washed pellets were resuspended in phosphate-buffered saline (PBS) containing RNase A (1 μg/1 μL) and Propidium Iodide (1 μg/1 μL). The incubation was carried for 30 min at room temperature in a dark environment. Samples were analyzed for emission in the PE-Texas Red channel using BD LSR Fortessa (BD Biosciences, San Jose, CA, USA) and by BD FACS Diva software. 10^4^ events for each sample were acquired in all analyses.

### RNA isolation, RT-PCR and qPCR

Total RNA was isolated from cells, quantified (NanoDrop spectrophotometer, Life Technologies, Carlsbad, CA, USA) and reverse transcribed according to the manufacturer’s instructions. Specific primers pairs used for RT-PCR and qPCR assays were designed using Oligo 4.0. and listed in Table 1. Semiquantitative PCR and qPCR assays were performed according to manufacturer’s instructions for Bio-Rad T100 thermal cycler and CFX Connect Real Time system (Biorad, Hercules, CA, USA), respectively. Relative gene expression quantification was measured by 2^−ΔΔCt^ method normalizing for the reference sample using Rps23 (Ribosomal Protein S23) as housekeeping gene.

**Table 1:**
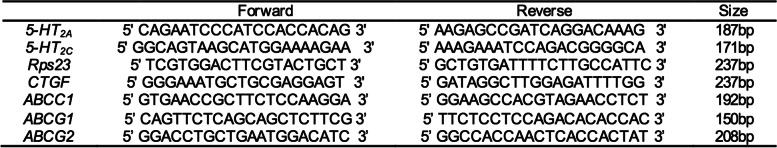
Primer pairs for qPCR

### Western Blot

RIPA buffer (Promega, Madison, Wisconsin, USA) was used for proteins’ extraction. Lysates (50–80 mg protein/sample) were blotted with anti-5HT_2C_ (1:500). Total lysates were normalized using anti-Vinculin (1:10000). The autoradiographs shown were obtained by ECL kit (Bio-Rad, Hercules, CA, USA).

### Statistical analysis

All the statistical analyses were carried out using GraphPad Prism 7. Kruskal Wallis test followed by Dunn’s correction was applied for multiple comparisons. Wilcoxon signed rank tests was assessed for comparison to a hypothetical value. Mann-Whitney test was used for pairwise comparisons. *P*-value<0.05 was considered statistically significant.

## Results

### Synthesis and in vitro receptor binding of serotoninergic receptor ligands

**Fig. 1:**
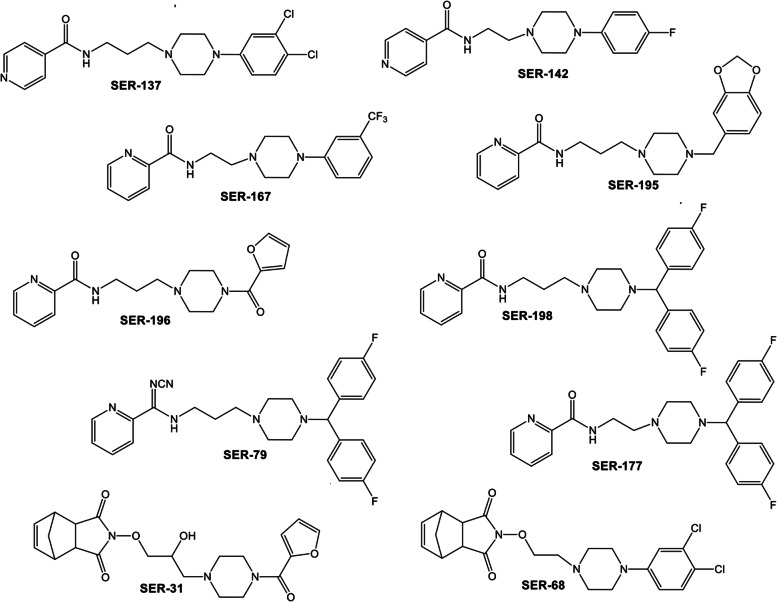
Chemical structures of serotoninergic receptor ligands (SER)

The synthetic strategy used for SER preparation (Fig. 1) was previously described [22–25]. Synthesized SER showed affinities in the nanomolar range towards 5-HT_1A_, 5-HT_2A_ and 5-HT_2C_ receptors (Table 2). Besides the outstanding 5-HT_2A_ receptor affinity and selectivity of compound SER142 (0.046 nM), other interesting Ki values were those of compounds SER137 (1.07 nM), SER196 (1.68 nM), SER195 (45.3 nM), SER167 (48.5 nM), and SER198 (77.8 nM) a picolinic derivative linked to bis(4-fluorophenyl) methyl piperazine moiety through to a propyl chain spacer. Instead, the analogue derivative characterized by a shorter ethyl chain spacer (SER177) showed a favorable affinity profile for 5-HT_2C_ receptors with Ki value of 0.8 nM. Other interesting Ki values towards this receptor, were those of compound SER68 characterized by 3,4-dichlorophenyl group as N-4 piperazine substituent, linked through an ethyl chain to a norbornene fragment that conferred affinity and selectivity toward 5-HT_2C_ receptor with Ki value of 1.13 nM. Instead, the norbornene derivative 4-[3-[4-(2-furoyl)piperazin-1-yl]propoxy-2-ol]-4-aza-tricyclo [5.2.1.02,6]dec-8-ene-3,5-dione (SER31) characterized by 2-hydroxy-propyl spacing unit was one of the most selective compound for the 5-HT_2C_ receptor with Ki = 5.04 nM. Moreover, the N′-cyanopicolinamidine derivative SER79, characterized once again by the bis(4-fluorophenyl) methyl piperazine moiety, showed affinity in the nanomolar range towards 5-HT_2C_ receptor (Ki = 21.4 nM) and weak or no affinity towards 5-HT_2A_ and 5-HT_1A_ receptors respectively.

**Table 2:**
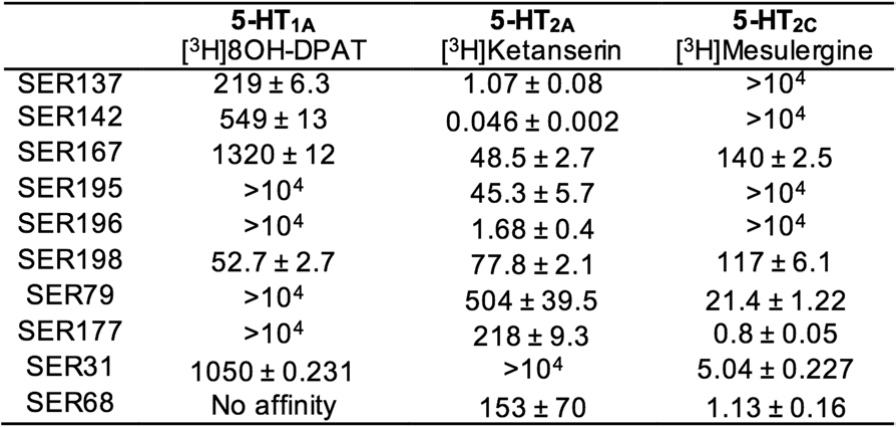
Affinities of SER for 5-HT1A, 5-HT2A and 5-HT2C receptors (Ki ± SD; nM)

### Antiproliferative effect of serotoninergic receptor ligands on BC cell lines

**Fig. 2:**
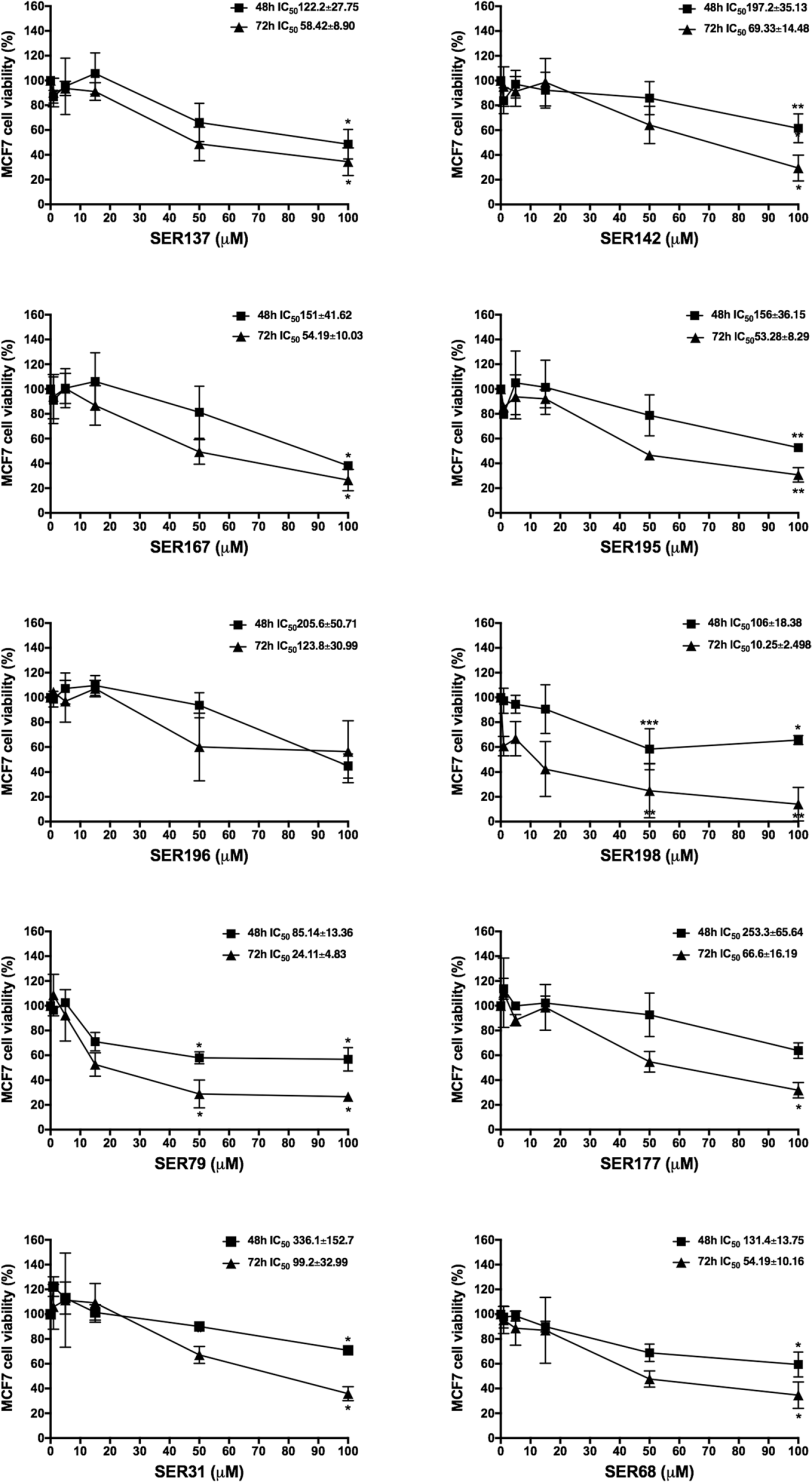
Effect of SER on MCF7 cell viability. MCF7 cells were treated with raising concentration (1 μM, 5 μM, 15 μM, 50 μM, 100 μM) of SER137, SER142, SER167, SER195, SER196, SER198, SER79, SER177, SER31, SER68. After 48 and 72 h, cell viability was assessed by sulforhodamine B assay (see Methods). The results were reported as percentage of viable cells compared to positive control (untreated cells), considered as maximum viability (100%). Data represent the mean ± SD of at least five independent triplicate experiments. * denotes statistically significant values compared with positive control (*adjp<0.05,**adjp<0.01,***adjp<0.001)

SER137, SER142, SER167, SER195, SER196, SER198, SER79, SER177, SER31, SER68, endowed with different binding affinity for 5-HT_2A_ and 5-HT_2C_ receptors, were examined for their ability to affect BC cell viability. MCF7 (ER^+^, PR^+^, HER2^−^) cells were treated with raising concentration (1 μM, 5 μM, 15 μM, 50 μM, 100 μM) of SER, in agreement with previous report [32]. We found that all compounds determined a dose-dependent growth inhibition upon both 48- and 72-h treatment. None effect of vehicle (DMSO) on cell viability was observed (Fig. S1a). All SER at 100 μM dose (except for SER196) significantly reduced MCF7 cell viability upon 72 h (60 to 80%, adjp<0.05). Of note, the effectiveness of SER79 and SER198 on reducing MCF7 cell viability was also observed at lower dose 50 μM (≈70%; adjp<0.05). Consistently, IC_50_ values were 24.11 μM and 10.25 μM for SER79 and SER198, respectively. For the other compounds IC_50_ ranged between 53.28 μM and 99.2 μM. Finally, IC_50_ value estimated for SER196 was 123.8 μM, out of concentration range tested (Fig. 2).

**Fig. 3:**
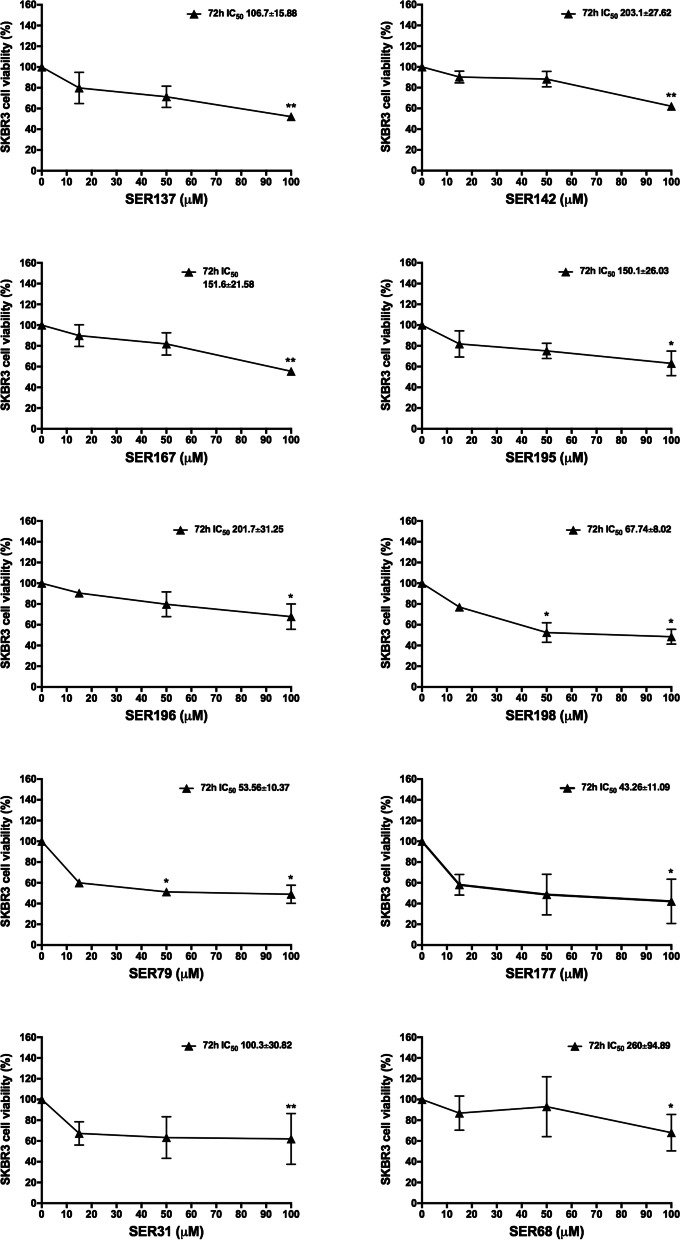
Effect of SER on SKBR3 cell viability. SKBR3 cells were treated with raising concentration (15 μM, 50 μM, 100 μM) of SER137, SER142, SER167, SER195, SER196, SER198, SER79, SER177, SER31, SER68. Cell viability was assessed, after 72 h, by sulforhodamine B assay (see Methods). The results were reported as percentage of viable cells compared to positive control (untreated cells), considered as maximum viability (100%). Data represent the mean ± SD of at least three independent triplicate experiments. * denotes statistically significant values compared with positive control (*adjp<0.05,**adjp<0.01)

**Fig. 4:**
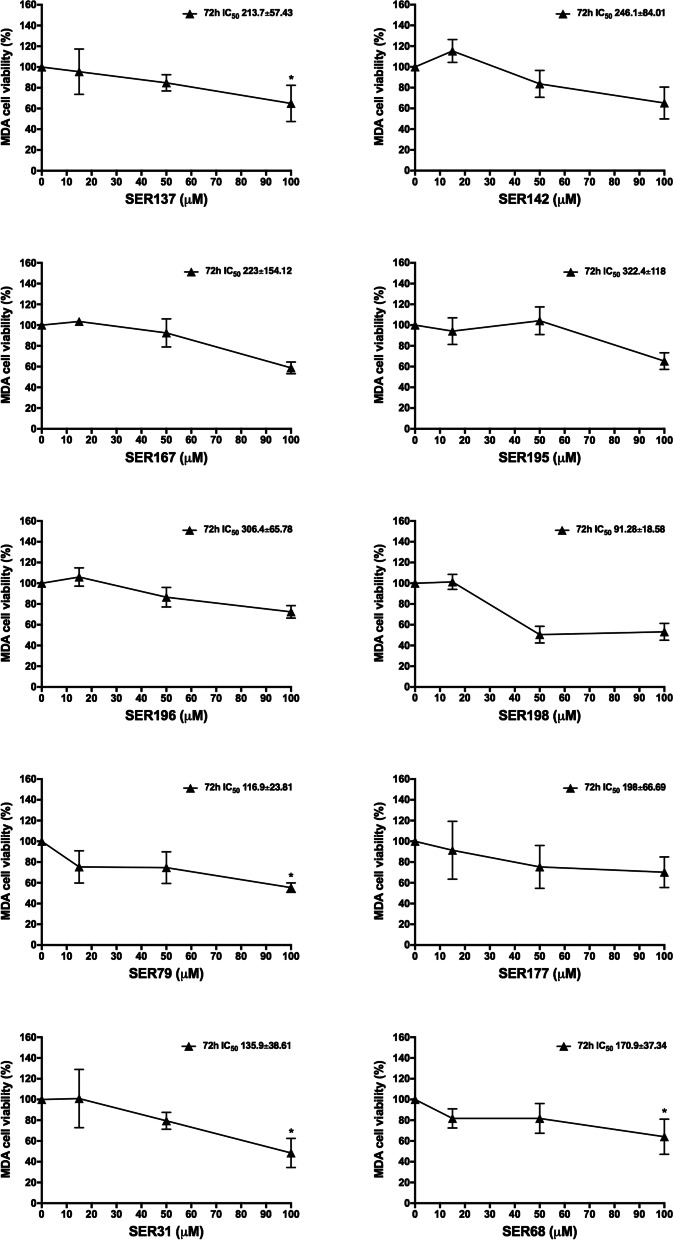
Effect of SER on MDA-MB231 cell viability. MDA cells were treated with raising concentration (15 μM, 50 μM, 100 μM) of SER137, SER142, SER167, SER195, SER196, SER198, SER79, SER177, SER31, SER68. Cell viability was assessed, after 72 h, by sulforhodamine B assay (see Methods). The results were reported as percentage of viable cells compared to positive control (untreated cells), considered as maximum viability (100%). Data represent the mean ± SD of at least three independent triplicate experiments. * denotes statistically significant values compared with positive control (*adjp<0.05)

We also evaluated the impact of SER137, SER142, SER167, SER195, SER196, SER198, SER79, SER177, SER31, SER68 on BC cell lines with different molecular features: SKBR3 (ER^−^, PR^+^, HER2^+^) and MDA-MB231 (ER^−^, PR^−^, HER2^−^). We observed that all SER at 100 μM dose significantly reduced SKBR3 cell viability. On the other hand, 50 μM SER79 and SER198 were able to significantly reduce SKBR3 cell viability (≈50%; adjp<0.05; Fig. 3). None effect of vehicle was observed (Fig. S1b). Such results highlighted that SER79 and SER198 were the most effective in reducing not only MCF7 but also SKBR3 cell viability. Of note, IC_50_ value estimated for SER79 and SER198 in SKBR3 cells was 53.56 μM and 67.74 μM, respectively. In parallel, we evaluated the effect of SER on triple negative MDA-MB231 cells. We found that only SER137, SER79, SER31, SER68 at 100 μM dose were able to inhibit triple negative MDA-MB231 cell growth. In the same condition, none effect of vehicle was observed (Fig. S1c). Notably, IC_50_ value estimated for SER79 in MDA-MB231 cells was 116.9 μM, higher than those obtained for both SKBR3 and MCF7 cells (Fig. 4). IC_50_ values estimated for SER in MCF7, SKBR3 and MDA-MB231 cells were listed in Table 3.

**Table 3:**
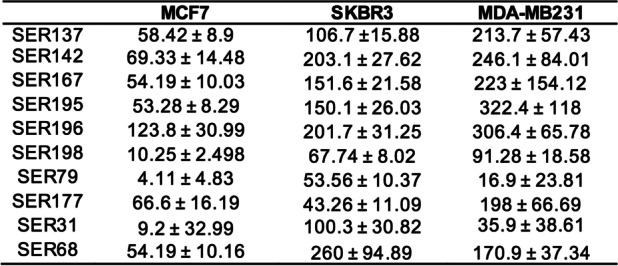
IC_50_ values (μM) of SER in MCF7, SKBR3 and MDA-MB231 BC cell lines

**Fig. 5:**
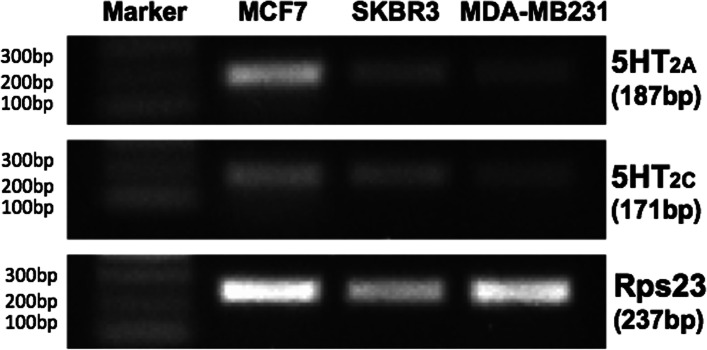
Representative gel images of RT-PCR assays for the 5-HT_2A_ and 5-HT_2C_ genes in MCF7, SKBR3 and MDA-MB231 cell lines. Rps23 was used as reference gene. Images have been cropped to improve the clarity of presentation

We also verified that the different effect of SER on BC cells was not attributable to a lack of expression of 5-HT receptors in SKBR3 and MDA-MB231 cells. Indeed, HT_2A_ and HT_2C_ receptors were detected in all cell lines suggesting that the different effect of SER on MCF7 (ER^+^, PR^+^, HER2^−^), SKBR3 (ER^−^, PR^+^, HER2^+^) and MDA-MB231 (ER^−^, PR^−^, HER2^−^) cells should be attributable to their different molecular features, which also give them a different degree of aggressiveness (Fig. 5).

### Effect of serotoninergic receptor ligands on MCF7 cell responsiveness to Tamoxifen

**Fig. 6:**
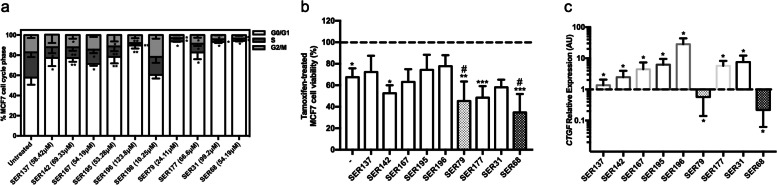
Effect of SER on MCF7 cell cycle and Tamoxifen response. **a** MCF7 cells were treated with SER137, SER142, SER167, SER195, SER196, SER198, SER79, SER177, SER31, SER68 at IC_50_ doses. After 72 h, cell cycle was assessed by Propidium Iodide staining (see Methods). The results were reported as percentage of cells in G0/G1, S and G2/M cell cycle phase. **b** Estrogen-starved MCF7 cells (48 h) were treated with Tamoxifen (5 μM) in presence of E_2_ (100nM) and selected SER (SER137, SER142, SER167, SER195, SER196, SER79, SER177, SER31, SER68) at IC_50_ dose. As positive control, the cells were treated with E_2_ and SER alone (without Tamoxifen, dotted line). After 72 h, cell viability was assessed by sulforhodamine B assay (see Methods). The results were reported as percentage of viable cells compared to positive control, considered as maximum viability (100%). **c** Estrogen-starved MCF7 cells (48 h) were treated with selected SER at IC_50_ doses in presence of E_2_ (100nM). After 72 h, mRNA levels of CTGF were determined by qPCR (see Methods and Table 1). Data were normalized on Ribosomal Protein S23 (Rps23) gene as internal standard and were reported as CTGF mRNA levels in MCF7 treated with SER relative to those in untreated cells (dotted line). **a**-**c** Data represent the mean ± SD of at least three independent triplicate experiments. * denotes statistically significant values compared with (**a**) percentage of untreated cells in G0/G1, S and G2/M cell cycle phase (*pval<0.05,**pval<0.01), (**b**) positive control (*adjp<0.05,**adjp <0.01,***adjp<0.001), (**c**) untreated cells (pval<0.05); # denotes statistically significant values compared with cells treated with Tamoxifen in absence of SER (#pval<0.05)

The effect of SER on cell viability was further investigated by analyzing cell cycle. At first, we observed that all SER, except for SER198, at dose corresponding to IC_50_ values, were able to induce cell cycle perturbation in MCF7 cells, causing a significant increase in the percentage of cells in G0/G1 phase, paralleled by a decrease of S phase (pval<0.05). Thus, all SER, except for SER198, affected cell viability and perturbed cell cycle (Figs. 2 and 6a). Therefore, we investigated their effect on MCF7 cell responsiveness to Tamoxifen by treating estrogen-starved cells with Tamoxifen and E2 in presence of SER. Notably, SER79 and SER68 further decreased cell viability compared with cells treated with Tamoxifen alone (≈15% with SER79 and ≈30% with SER68; pval<0.05; Fig. 6b). Then, we analyzed *CTGF* mRNA levels in MCF7 cells treated with SER. Interestingly, we found that in presence of SER79 and SER68, while not of the other compounds, *CTGF* mRNA levels were significantly lower compared with those in untreated cells (≈60% with SER79 and ≈80% with SER68; pval<0.05; Fig. 6c). Of note, we found that none of SER compounds (IC_50_ values) significantly affected SKBR3 and MDA-MB231 cell cycle. In addition, *CTGF* was also expressed in SKBR3 and MDA-MB231 cells and, at variance with MCF7 cells, no significant SER-induced change was observed (Fig. S2).

**Fig. 7:**
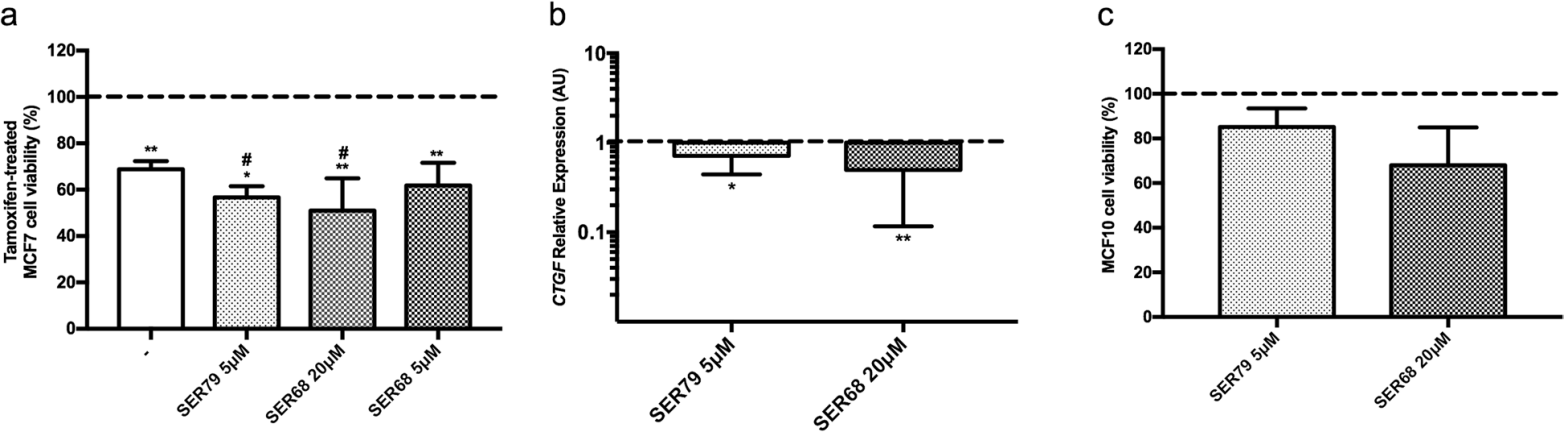
Effect of low doses of SER79 and SER68 on MCF7 and MCF10A cells. **a** Estrogen-starved MCF7 cells (48 h) were treated with Tamoxifen (5 μM) in presence of E_2_ (100nM) and SER79 (5 μM) or SER68 (20 μM, 5 μM). As positive control, the cells were treated with E_2_ and SER alone (without Tamoxifen, dotted line). After 72 h, cell viability was assessed by sulforhodamine B assay (see Methods). The results were reported as percentage of viable cells compared to positive control, considered as maximum viability (100%). **b** Estrogen-starved MCF7 cells (48 h) were treated with SER79 (5 μM) or SER68 (20 μM) in presence of E_2_ (100 nM). After 72 h, mRNA levels of *CTGF* were determined by qPCR (see Methods and Table 1). Data were normalized on Ribosomal Protein S23 (Rps23) gene as internal standard. Bars represent *CTGF* mRNA levels in MCF7 treated with SER relative to those in untreated cells (dotted line). **c** MCF10A non-cancer breast epithelial cells were treated with SER79 (5 μM) or SER68 (20 μM). After 72 h, cell viability was assessed by sulforhodamine B assay (see Methods). The results were reported as percentage of viable cells compared to untreated cells (dotted line), considered as maximum viability (100%). **a**-**c** Data represent the mean ± SD of at least three independent triplicate experiments. * denotes statistically significant values compared with (**a**) positive control (*adjp<0.05,**adjp<0.01), (**b**, **c**) untreated cells (*pval<0.05, *pval<0.01); # denotes statistically significant values compared with cells treated with Tamoxifen in absence of SER (#pval<0.01)

To further study the effectiveness of SER79 and SER68 in ameliorating MCF7 responsiveness, we treated the cells with Tamoxifen in presence of SER doses lower than IC_50_ values (5 μM SER79; 20 μM or 5 μM SER68). We found that 5 μM SER79 and 20 μM SER68 further decreased cell viability compared with cells treated with Tamoxifen alone (≈15% with SER79 and ≈20% with SER68; pval<0.01; Fig. 7a). No further effect was elicited by 5 μM SER68. Interestingly, we observed that both 5 μM SER79 and 20 μM SER68 were able to significantly reduce CTGF expression (≈30% with SER79 and ≈50% with SER68; pval<0.05; Fig. 7b). We also tested the effect of these compounds on non-cancer breast epithelial cells MCF10A. No effect was detected on cell viability (Fig. 7c). Overall, these data suggested that SER79 and SER68 improve Tamoxifen responsiveness of MCF7 cells without affecting non-cancer cells (Fig. 7).

### Effect of SER on Tamoxifen responsiveness of drug-resistant MCF7 cells

**Fig. 8:**
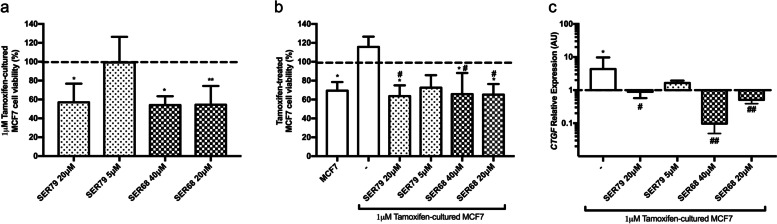
Effect of SER79 and SER68 on Tamoxifen-cultured MCF7 cells. MCF7 cells were cultured in presence of Tamoxifen (1 μM) for 10 days. Estrogen-starved MCF7 and Tamoxifen-cultured MCF7 cells (48 h) were (**a**-**c**) treated with SER79 (20 μM, 5 μM) or SER68 (40 μ, 20 μM), (**b**) treated with Tamoxifen (5 μM) in presence of SER79 (20 μM, 5 μM) or SER68 (40 μM, 20 μM). All treatments were carried out in presence of E_2_ (100 nM) for 72 h. As positive control, the cells were treated with (**a**-**c**) E_2_ alone or (**b**) E_2_ in absence or in presence of SER (as indicated, dotted line). **a**,**b** Cell viability was assessed by sulforhodamine B assay (see Methods). The results were reported as percentage of viable cells compared to positive control, considered as maximum viability (100%). **c** mRNA levels of *CTGF* were determined by qPCR (see Methods and Table 1). Data were normalized on Ribosomal Protein S23 (Rps23) gene as internal standard and reported as *CTGF* mRNA levels in Tamoxifen-cultured MCF7 relative to those in MCF7 cells (dotted line). **a**-**c** Graphs represent the mean ± SD of at least three independent triplicate experiments. * denotes statistically significant values compared with (**a**,**b**) positive control (*adjp<0.05, ** adjp<0.01) or (**c**) MCF7 cells (*pval<0.05); # denotes statistically significant values compared with (b) cells treated with Tamoxifen in absence of SER or (**c**) untreated Tamoxifen-cultured MCF7 cells (#pval<0.01, ##pval<0.0001)

To further investigate the effect of SER79 and SER68 on Tamoxifen responsiveness and *CTGF* expression in MCF7 cells, we obtained - by a continuous treatment of 10 days with 1 μM Tamoxifen – a cellular model less sensitive to the drug (Tamoxifen-cultured MCF7). We observed that 20 μM SER79, 20 μM and 40 μM SER68, while not 5 μM SER79, reduced viability of Tamoxifen-cultured MCF7 cells by about 40% (adjp<0.01; Fig. 8a). Tamoxifen treatment alone did not affect viability of these cells. However, in the presence of 20 μM SER79, 20 μM and 40 μM SER68, Tamoxifen elicited a further 40% reduction of viability, similar to that achieved in Tamoxifen-responsive MCF7 cells (adjp<0.01; Fig. 8b). No Tamoxifen effect was observed in the presence of 5 μM SER79 (Fig. 8b). In parallel, *CTGF* mRNA levels were significantly increased in Tamoxifen-cultured MCF7 cells (pval<0.001; Fig. 8c). Both SER68 and SER79 – at doses able to restore Tamoxifen responsiveness - significantly reduced *CTGF* to levels similar (for SER79) or significantly lower (for SER68) than those detected in Tamoxifen-responsive MCF7 cells (pval<0.05; Fig. 8c).

**Fig. 9:**
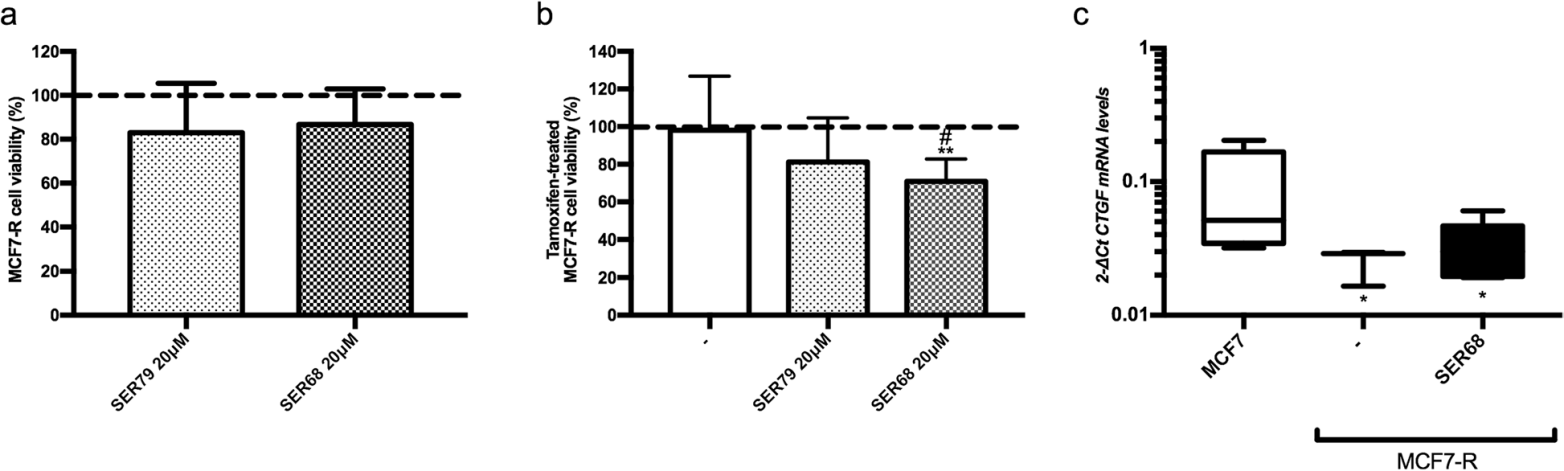
Effect of SER79 and SER68 on Tamoxifen-resistant (MCF7-R) cells. **a** Estrogen starved MCF7-R were treated with SER79 (20 μM, 5 μM) or SER68 (20 μM) in presence of E_2_ (100 nM). **b** Estrogen-starved MCF7-R were treated with Tamoxifen (5 μM) in presence of E_2_ and SER79 (20 μM) or SER68 (20 μM). **a,b** As positive control, the cells were treated with E_2_ alone, in absence or in presence of SER (dotted line). After 72 h, cell viability was assessed by sulforhodamine B assay (see Methods). The results were reported as percentage of viable cells compared to positive control, considered as maximum viability (100%). **c** mRNA levels of *CTGF* were determined by qPCR (see Methods and Table 1) in MCF7-R cells upon treatment with SER68 (20 μM). Data were normalized on Ribosomal Protein S23 (Rps23) gene as internal standard and reported as *CTGF* mRNA levels in MCF7-R. **a**-**c** Graphs represent the mean ± SD of at least three independent triplicate experiments. * denotes statistically significant values compared with (**b**) positive control or (**c**) MCF7 cells (*adjp<0.05; **adjp<0.001). # denotes statistically significant values compared with untreated MCF7-R (#pval<0.05)

Next, we obtained Tamoxifen-resistant MCF7 cells (MCF7-R; see Materials and Methods). As reported in Supplementary Fig. 3, Tamoxifen did not reduce MCF7-R cell viability up to 6 mM. Moreover, Tamoxifen-treated MCF7-R cells displayed higher mRNA levels of ABCC1, ABCG1 and ABCG2, markers of multidrug resistance [32] (Fig. S3). If used alone, neither 20 μM SER79 neither 20 μM SER68 had effect onto MCF7-R cells (Fig. 9a). In co-treatment with 5 μM Tamoxifen, SER68 significantly reduced viability of MCF7-R cells (pval<0.01; Fig. 9b). No significant effect was achieved by co-treatment with Tamoxifen and SER79 (Fig. 9b). At variance with Tamoxifen-cultured cells, MCF7-R cells displayed a significant reduction of *CTGF* levels compared with Tamoxifen-responsive MCF7 cells (adjp<0.05; Fig. 9c). However, treatment with SER68 did not further reduce *CTGF* mRNA content. (Fig. 9).

Finally, we verified that the effect of SER79 and SER68 on MCF7, either responsive or resistant to Tamoxifen was not attributable to changes of expression of HT_2C_ receptor. As shown in Supplementary Fig. 4, no difference in protein levels of HT_2C_ receptors was detected upon SER treatment in all cell types (Fig. S4).

## Discussion

New biological insights highlighted a role of serotonin in virtually all major organs outside the central nervous system [33]. Thus, 5-HT has numerous important peripheral functions in humans [18]. Among them, it is integral part of mammary epithelial homeostatic system in ensuring normal tissue function and becomes dysregulated in human breast tumor [34, 35]. 5-HT signaling has been related with cancer cell growth, differentiation, angiogenesis and metastasis, suggesting an association between its levels and tumor aggressiveness and/or prognosis [15, 18, 35]. Physiological responses to serotonin include both tumor-suppressing and tumor-promoting activities. Indeed, while controlling homeostatic regulatory mechanisms in normal mammary epithelium, 5-HT signaling appears to favor malignant progression of human BC [17]. Differences in the components of serotonin system, including the ability to synthesize 5-HT and/or specific receptors, may explain these opposite effects [15, 17]. Of note, transcriptomic and metabolomic data from breast tumor specimens highlighted the correspondence between poor prognosis and increased tumor-specific serotonin production [36]. Seven distinct families of 5-HT receptors are expressed in a tissue-specific manner across a variety of normal and tumor cells [37]. In BC, serotonin confers proliferative advantage to tumor cells by increasing proliferation rate and decreasing programmed cell death, mainly through 5-HT_2A_ and 5-HT_2C_ receptors [18, 26].

We previously synthesized serotoninergic receptor ligands with high affinity (in the nanomolar range) and selectivity binding profile towards 5-HT_2A_ and 5-HT_2C_ receptors [22]. Here, we analyzed the effect of such compounds on BC cell survival. Interestingly, we observed that their different ability to affect MCF7 (ER^+^, PR^+^, HER2^−^), SKBR3 (ER^−^, PR^+^, HER2^+^) and MDA-MB231 (ER^−^, PR^−^, HER2^−^) BC cell growth was not due to a lack of expression of 5-HT_2_ receptors but eventually attributable to their different molecular features, which also give them a different degree of aggressiveness. In addition, considering that structural analogies exist among serotoninergic receptors and that SER were selective, while not exclusive, for binding 5-HT_2A_ and 5-HT_2C_, a possible involvement of other components of the serotoninergic receptor pattern, could not be excluded. It should also be noticed that several factors may influence the effect of SER on cell viability, including the ability to reach the receptor site and the intrinsic activity. Thus, the measure of receptor affinity not necessarily coincides to that of intrinsic activity of compounds. In line with this, even though the measure of receptor affinities were in nanomolar range, the effect of SER on cell viability has been observed at micromolar doses. This is also in agreement with a previous publication in a different cell type [37].

Interestingly, inhibition of cell viability in MCF7 cells is paralleled by cell cycle changes. For instance, upon treatment with different SER, MCF7 cells accumulate in G0/G1 phase and fail to proceed to S phase. Such effect does not occur in both SKBR3 and MDA-MB231. Hormone receptor-positive tumors obtain substantial benefit from treatment with Tamoxifen [20, 21]. We previously reported that Tamoxifen responsiveness of ER^+^ BC cells inversely correlates with Connective Tissue Growth Factor, providing additional clues to the hypothesis of its contribution to drug sensitivity in BC [31]. It has been described that inhibitors of serotoninergic pathway components reduced sphere-forming activity of breast tumor cell lines in dose-dependent fashion and synergized with docetaxel to shrink breast tumor xenografts [36]. In line with this concept, we provide evidence that some serotoninergic receptor ligands, namely SER79 and SER68, improve the effectiveness of Tamoxifen treatment on ER^+^ MCF7 BC cells modulating CTGF expression. CTGF may be triggered by serotonin and their association has been already described [38, 39].

Thus, our results identified new compounds able to target serotonin signaling, and in turn CTGF, and therefore potentially usable in combination with Tamoxifen improving its effectiveness on ER^+^ BC patients. However, CTGF levels are reduced in a cellular model of Tamoxifen resistance. In this same model, SER68 may restore Tamoxifen responsiveness, without further reducing CTGF levels. It should also be pointed out that SER68 and/or other SER exert an inhibitory action also on SKBR3 and MDA-MB231, although at a lower extent. Again, no modulation of CTGF levels have been detected in these cells, suggesting a potential involvement of ER and/or PR in SER-mediated effects on CTGF.

Adjuvant endocrine therapies may contribute to depression and anxiety in patients with cancer [40, 41]. Antidepressant medications, including antagonists of serotonin receptor and/or SSRIs, may be co-prescribed with Tamoxifen in BC [42]. However, some antidepressant agents interfere with Tamoxifen metabolism, compromising its efficacy [43]. In this regard, the use of SSRIs has been associated with increased tumor proliferative index in patients with late-stage BC compared to patients non-users of SSRIs [35]. Nevertheless, drug interactions involving Tamoxifen and antidepressant medications remain controversial [15, 23, 42]. The discovery of novel compounds directly targeting serotonin signaling may contribute to tailor new therapeutic strategies usable in combination with endocrine therapies, improving their efficacy for treating cancer patients.

## Conclusions

We identified serotoninergic receptor ligands able to target serotonin signaling, and in turn *CTGF*, also ameliorating the sensitivity to Tamoxifen in ER^+^ BC cells. Thus, they represent new compounds potentially usable in combination with Tamoxifen improving its effectiveness on ER^+^ BC patients.

## Supplementary Information

**Additional file 1: Figure S1.** Effect of vehicle on cell viability of (a) MCF7, (b) SKBR3 and (c) MDA-MB231.

**Additional file 2: Figure S2.** Effect of SER on SKBR3 and MDA-MB231 cells.

**Additional file 3: Figure S3.** Establishment of MCF7 Tamoxifen resistant (MCF7-R) cells.

**Additional file 4: Figure S4.** 5-HT2C protein in MCF7, Tamoxifen-cultured MCF7 and MCF-R cells in absence or in presence of SER.

**Additional file 5.**

## Data Availability

The datasets generated and/or analyzed during the current study are available from the corresponding author on reasonable request.

## Abbreviations

5-HT: 5-Hydroxytryptamine, Serotonin
ER^+^: Estrogen Receptor Positive
SER: Serotoninergic receptor ligands
BC: Breast Cancer
CTGF: Connective Tissue Growth Factor
SERMs: Selective Estrogen Receptor Modulators
SSRIs: Selective Serotonin Reuptake Inhibitors
E2: Estradiol
