# Supplementary material for: Serotoninergic receptor ligands improve Tamoxifen effectiveness on breast cancer cells
Source: BMC Cancer. 2022 Feb 15;22:171. doi: 10.1186/s12885-021-09147-y (PMC8845285; doi:10.1186/s12885-021-09147-y)
Supplement: Supplementary file 1 — Additional file 1: Figure S1. Effect of vehicle on cell viability of (a) MCF7, (b) SKBR3 and (c) MDA-MB231. [file 12885_2021_9147_MOESM1_ESM.pdf]

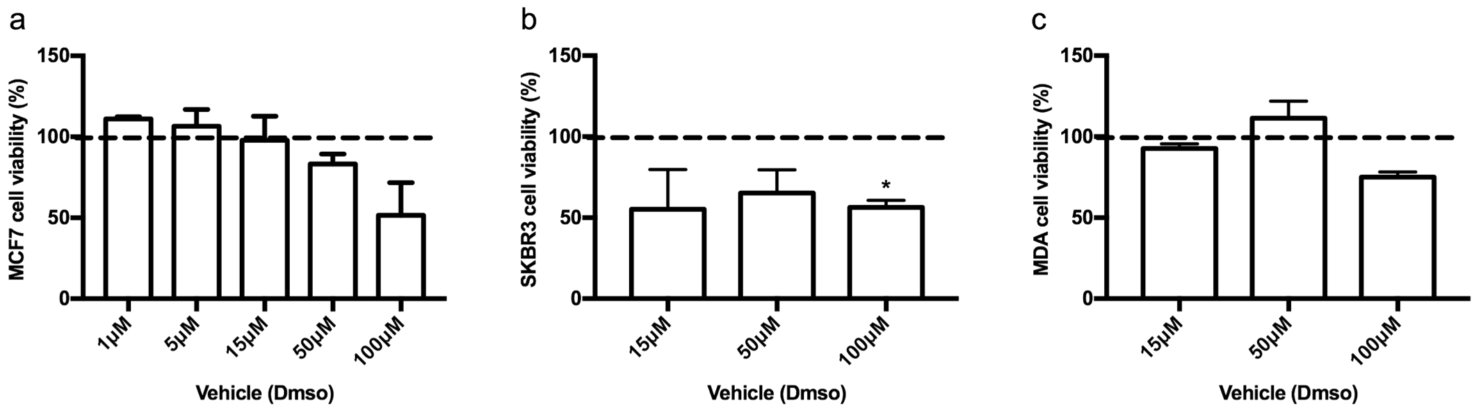

Figure S1. Effect of vehicle on cell viability of (a) MCF7, (b) SKBR3 and (c) MDA-MB231. MCF7, SKBR3 and MDA-MB231 cells were treated with SER vehicle (DMSO) at increasing doses (corresponding to those used for different SER concentrations; see Figure 2,3,4). After 72h, cell viability was assessed by sulforhodamine B assay (see Methods). The results were reported as percentage of viable cells compared to positive control (untreated cells; dotted line), considered as maximum viability (100%). Data represent the mean ± SD of at least three independent triplicate experiments. * denotes statistically significant values compared with positive control (*adjp<0.05).
