# Supplementary material for: Serotoninergic receptor ligands improve Tamoxifen effectiveness on breast cancer cells
Source: BMC Cancer. 2022 Feb 15;22:171. doi: 10.1186/s12885-021-09147-y (PMC8845285; doi:10.1186/s12885-021-09147-y)
Supplement: Supplementary file 2 — Additional file 2: Figure S2. Effect of SER on SKBR3 and MDA-MB231 cells. [file 12885_2021_9147_MOESM2_ESM.pdf]

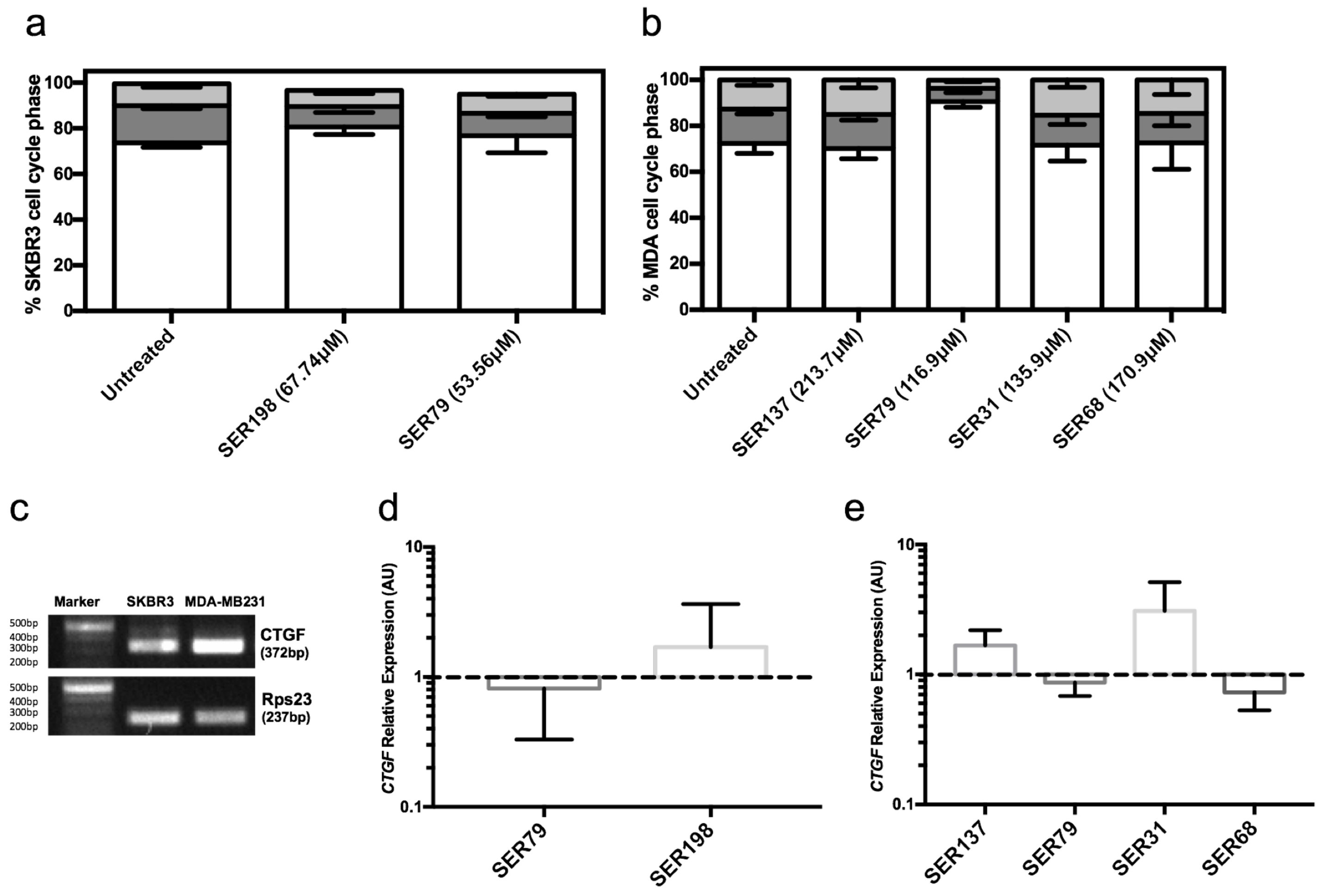

Figure S2. Effect of SER on SKBR3 and MDA-MB231 cells. (a-b) SKBR3 and MDA-MB231 cells were treated with selected SER at IC_{50} doses. After 72h, cell cycle was assessed by Propidium Iodide staining (see Methods). The results were reported as percentage of cells in G0/G1, S and G2/M cell cycle phase. (c) Representative gel images of RT-PCR assays for *CTGF* gene in SKBR3 and MDA-MB231 cell lines. Rps23 was used as reference gene. Images have been cropped to improve the clarity of presentation. (d) SKBR3 cells were treated with SER79 and SER198 (at IC_{50} doses). (e) MDA-MB231 cells were treated with SER137, SER79, SER31 and SER198 (at IC_{50} doses). (d-e) After 72h, mRNA levels of *CTGF* were determined by qPCR (see Methods and Table 1). Data were normalized on Ribosomal Protein S23 (Rps23) gene as internal standard. Bars represent the mean ± SD of at least three independent experiments and show *CTGF* mRNA levels in (d) SKBR3 and (e) MDA-MB231 treated with SER relative to those in untreated cells (dotted line).
