# Supplementary material for: Serotoninergic receptor ligands improve Tamoxifen effectiveness on breast cancer cells
Source: BMC Cancer. 2022 Feb 15;22:171. doi: 10.1186/s12885-021-09147-y (PMC8845285; doi:10.1186/s12885-021-09147-y)
Supplement: Supplementary file 3 — Additional file 3: Figure S3. Establishment of MCF7 Tamoxifen resistant (MCF7-R) cells. [file 12885_2021_9147_MOESM3_ESM.pdf]

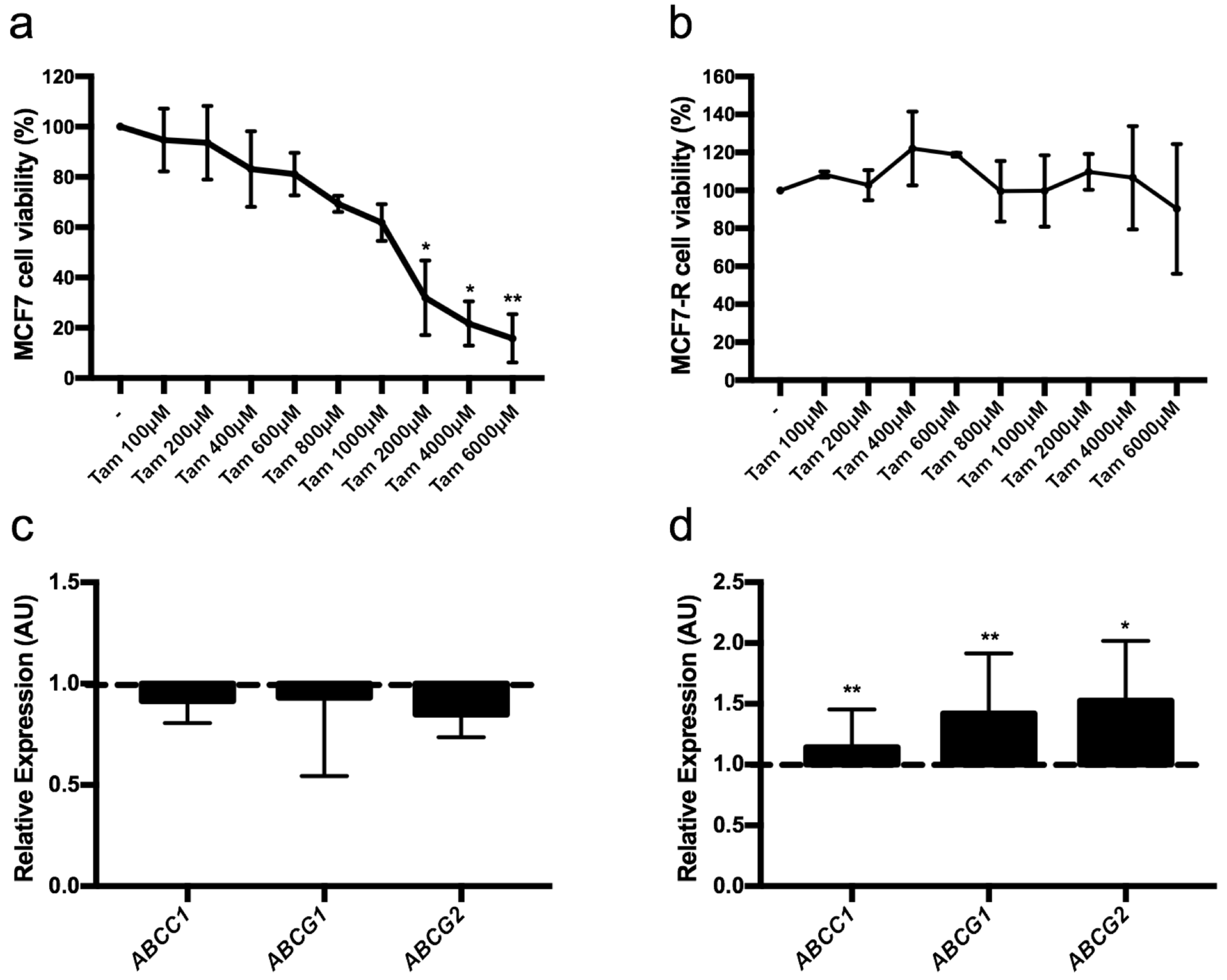

Figure S3. Establishment of MCF7 Tamoxifen resistant (MCF7-R) cells. MCF7 cells were cultured with Tamoxifen (1µM), in absence of E_2, for 4 months (MCF7-R). (a-b) MCF7 and MCF7-R cells were treated with increasing concentration of Tamoxifen (100nM to 6µM) in presence of E_2 (100nM) for 72h. As positive control, cells were treated with E_2 alone. Cell viability was measured by sulforhodamine B assay (see Methods). The results were reported as percentage of viable cells compared to untreated cells, considered as maximum viability (100%). (c-d) MCF7 and MCF7-R cells were treated with Tamoxifen (5µM) in presence of E_2 (100nM) for 72h. The expression levels of *ABCC1*, *ABCG1*, *ABCG2* were measured by qPCR (see Methods and Table 1). Data were normalized on Ribosomal Protein S23 (Rps23) gene as internal standard and represented as ABC transporters mRNA levels in (c) MCF7 and (d) MCF7-R treated with Tamoxifen compared to those in untreated cells (dotted line). Data represent the mean ± SD of at least three independent triplicate experiments. (a) * denotes statistically significant values compared with untreated cells (a:*adjp<0.05,**adjp<0.01; b: *pval<0.05,**pval<0.01).
