# Supplementary material for: Serotoninergic receptor ligands improve Tamoxifen effectiveness on breast cancer cells
Source: BMC Cancer. 2022 Feb 15;22:171. doi: 10.1186/s12885-021-09147-y (PMC8845285; doi:10.1186/s12885-021-09147-y)
Supplement: Supplementary file 4 — Additional file 4: Figure S4. 5-HT2C protein in MCF7, Tamoxifen-cultured MCF7 and MCF-R cells in absence or in presence of SER. [file 12885_2021_9147_MOESM4_ESM.pdf]

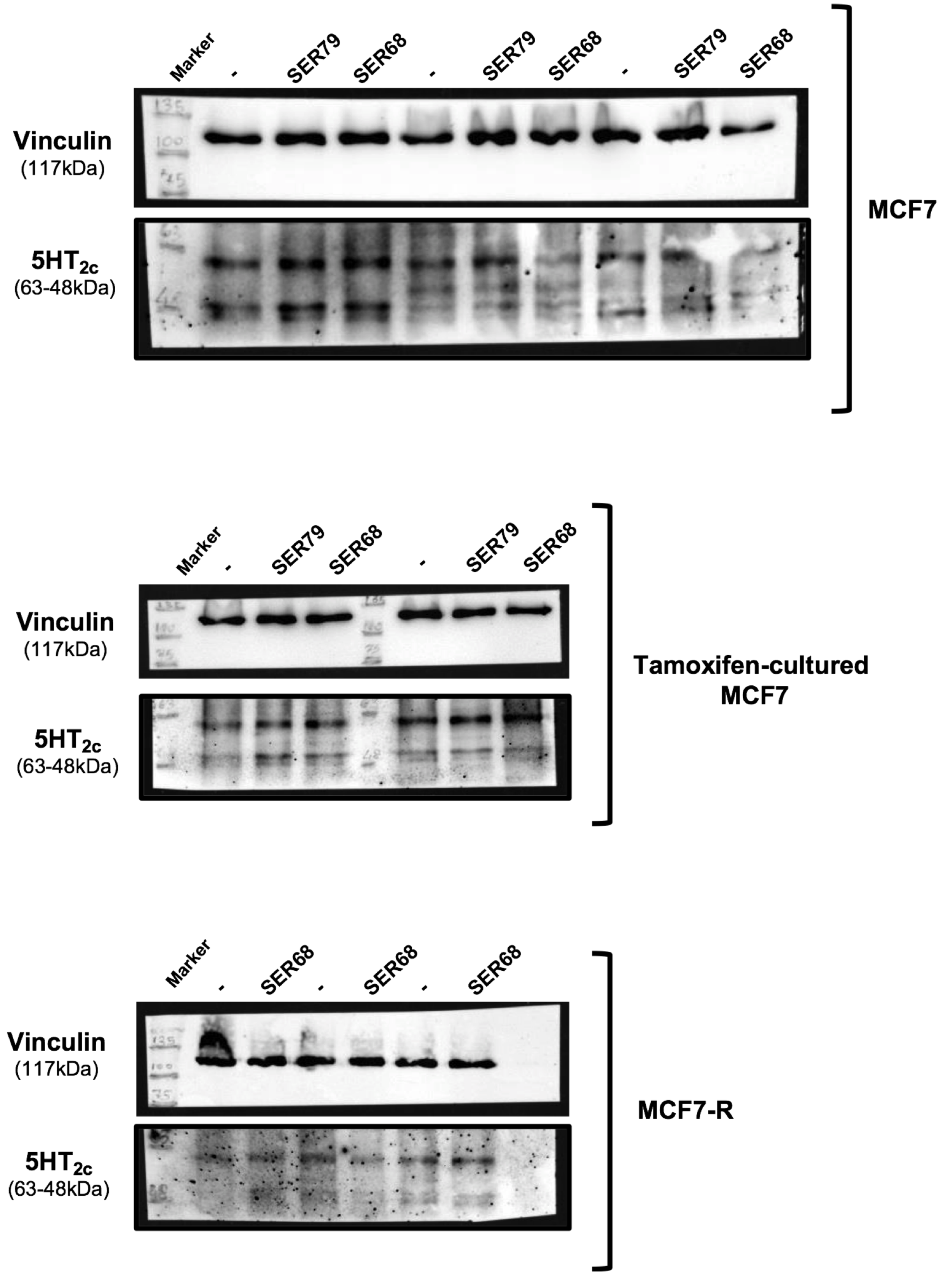

Figure S4. 5-HT_{2C} protein in MCF7, Tamoxifen-cultured MCF7 and MCF-R cells in absence or in presence of SER. Cells were treated with 5μM SER79 or 20μM SER68. Alter 72h, cell lysates were obtained and 5-HT_{2C} protein levels were measured by western blot analysis. Vinculin was used for protein levels normalization. Images have been cropped to improve the clarity of presentation and are representative gel images of at least two independent experiments.
