# Supplementary material for: Serotoninergic receptor ligands improve Tamoxifen effectiveness on breast cancer cells
Source: BMC Cancer. 2022 Feb 15;22:171. doi: 10.1186/s12885-021-09147-y (PMC8845285; doi:10.1186/s12885-021-09147-y)
Supplement: Supplementary file 5 — Additional file 5. [file 12885_2021_9147_MOESM5_ESM.pdf]

MCF7 Vinculin

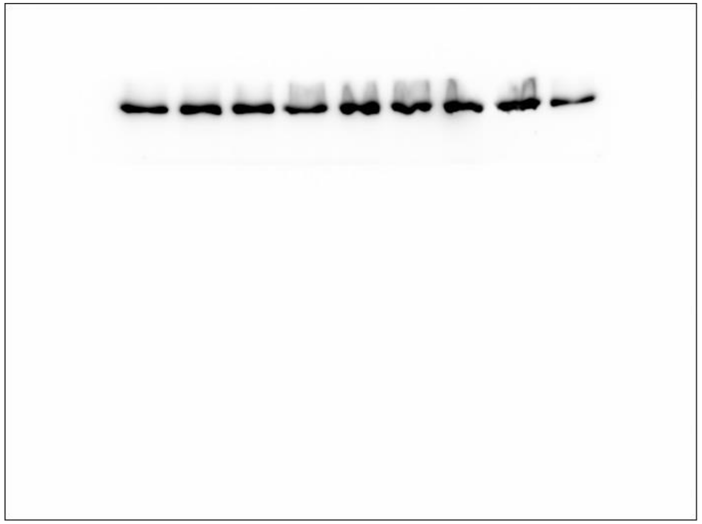

Chemiluminescence

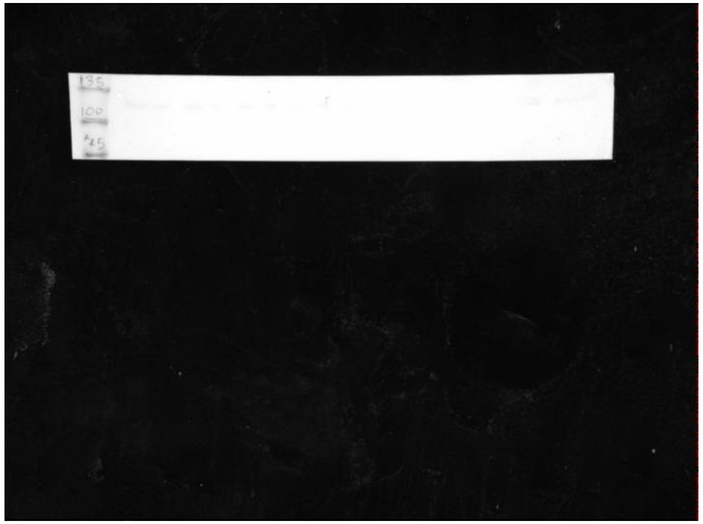

Original Blot

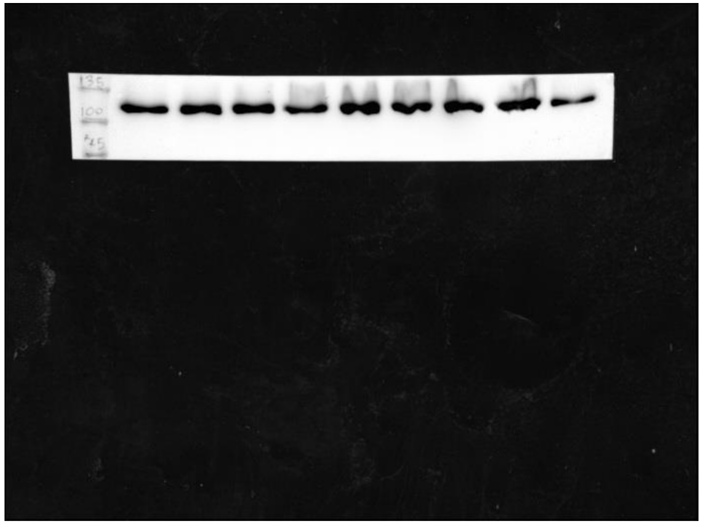

Merge

MCF7 5HT2C

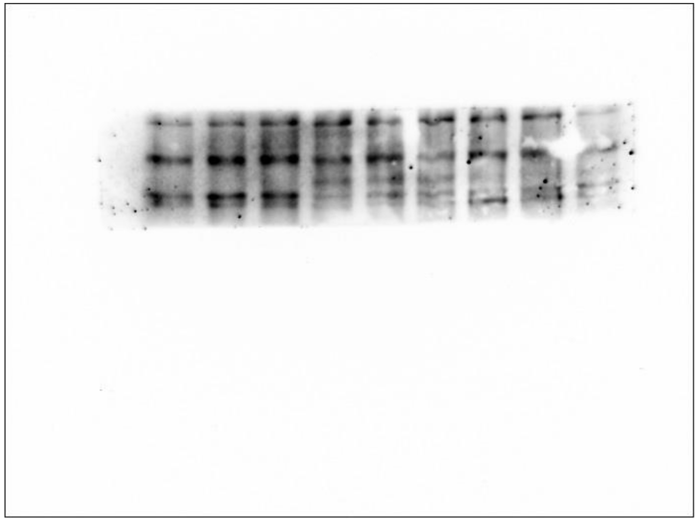

Chemiluminescence

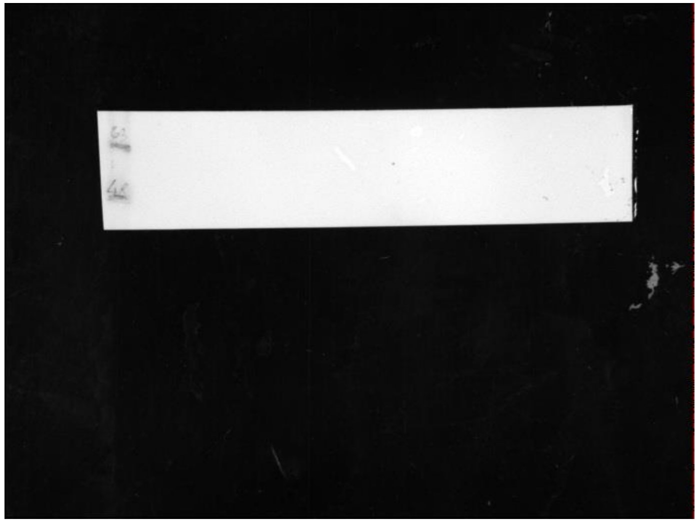

Original Blot

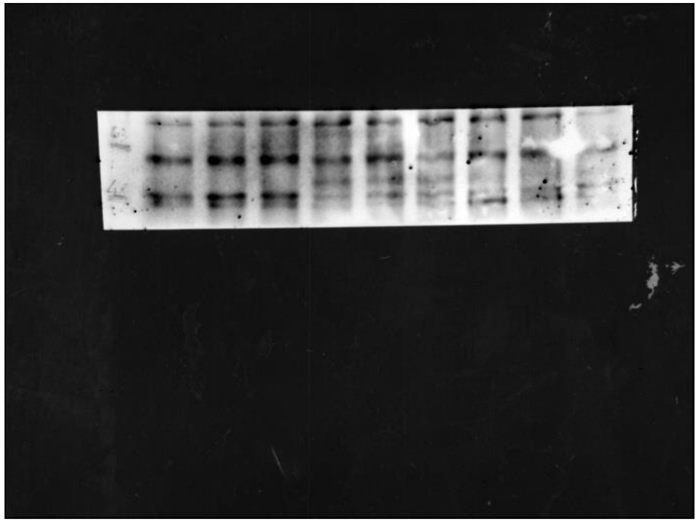

Merge

Tamoxifen-cultured MCF7 Vinculin

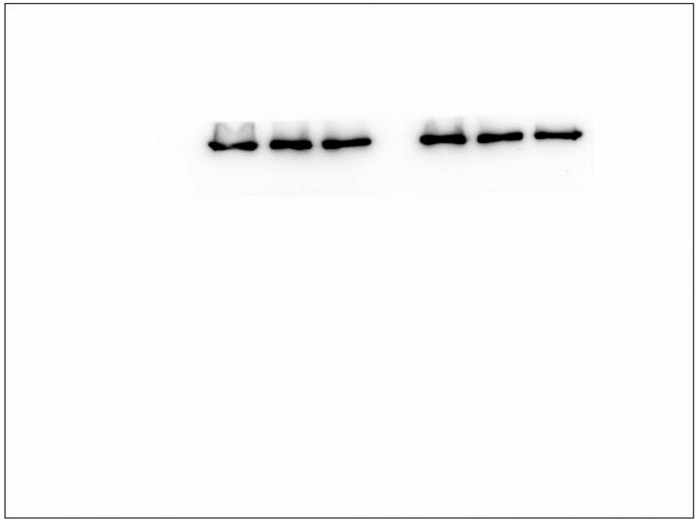

Chemiluminescence

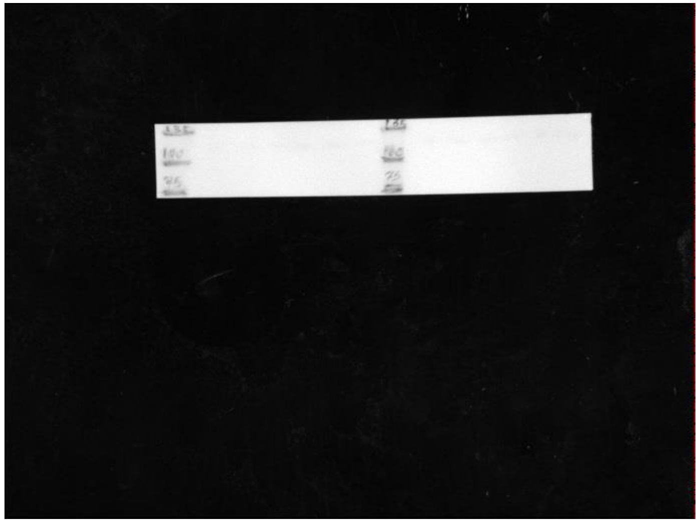

Original Blot

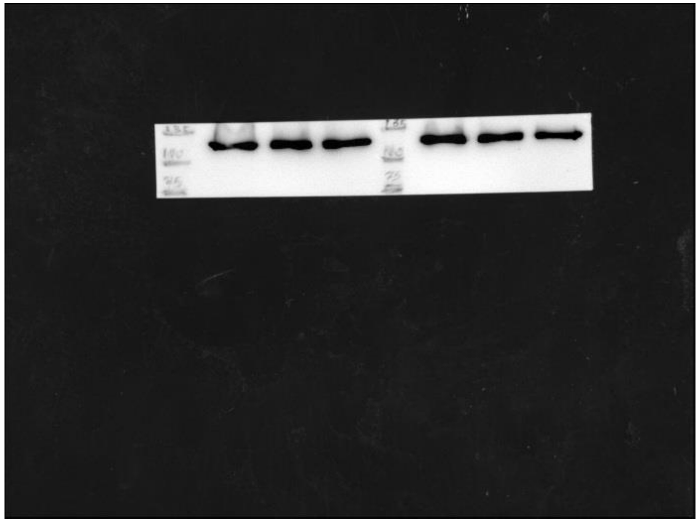

Merge

Tamoxifen-cultured MCF7 5HT2C

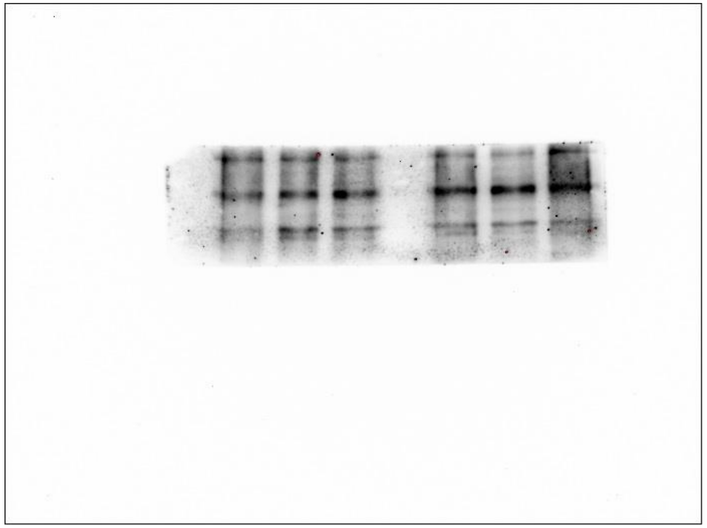

Chemiluminescence

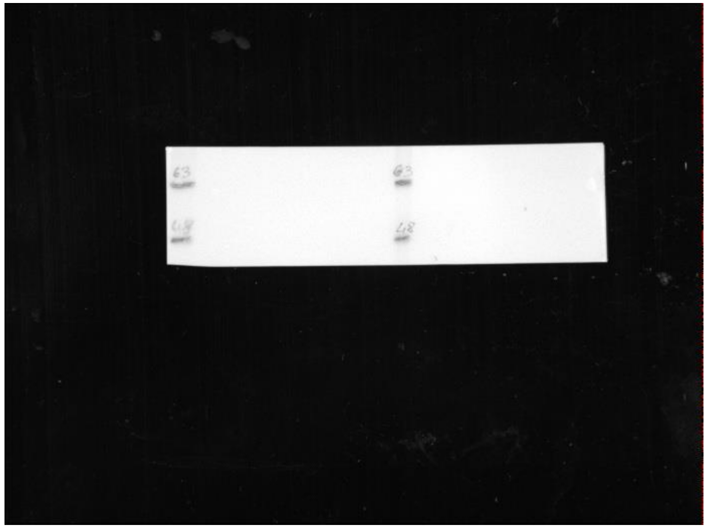

Original Blot

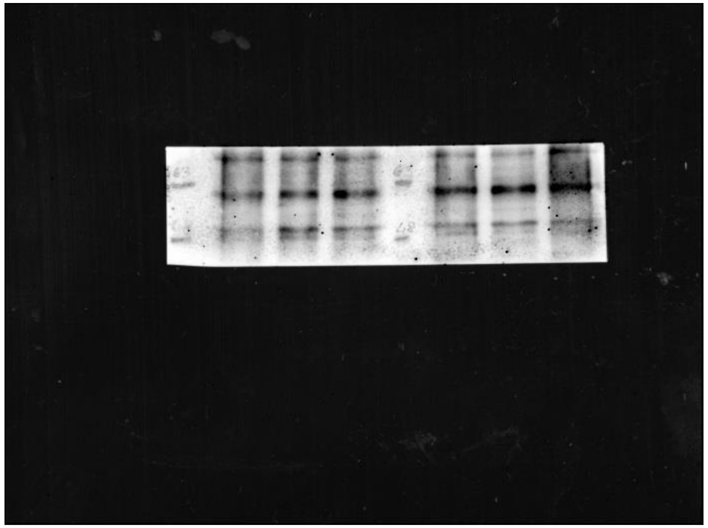

Merge

MCF7-R Vinculin

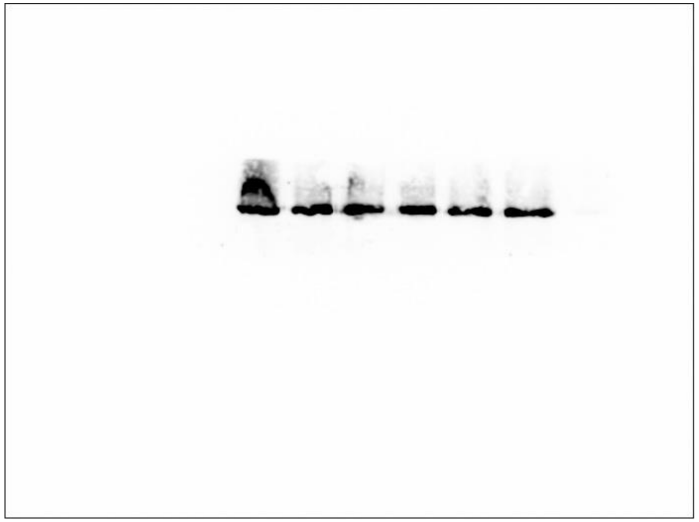

Chemiluminescence

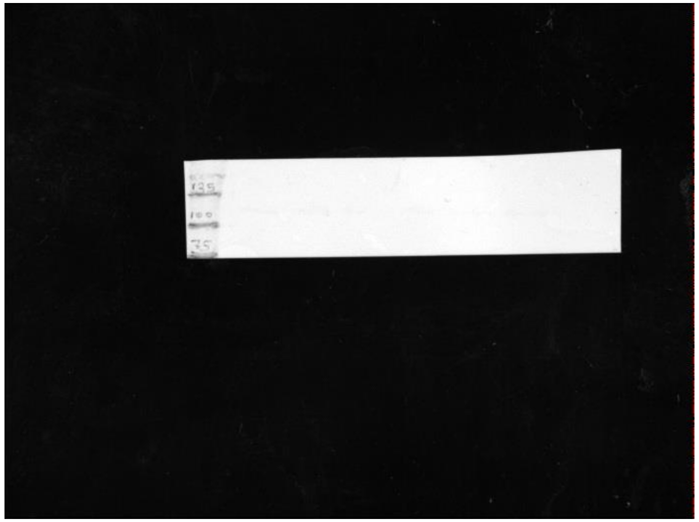

Original Blot

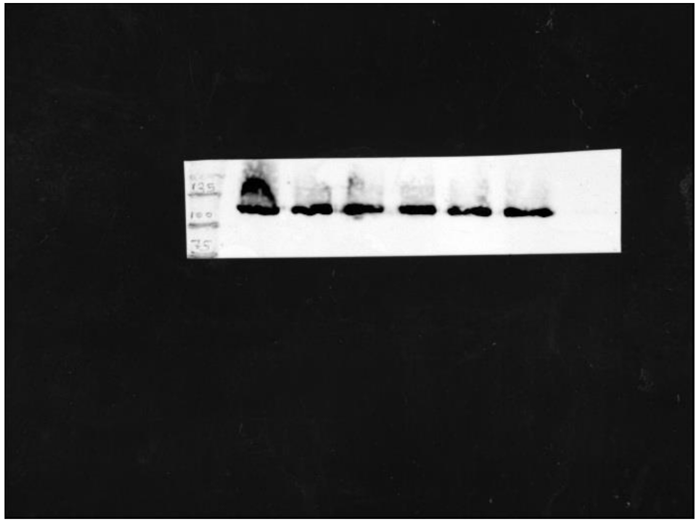

Merge

MCF7-R 5HT2C

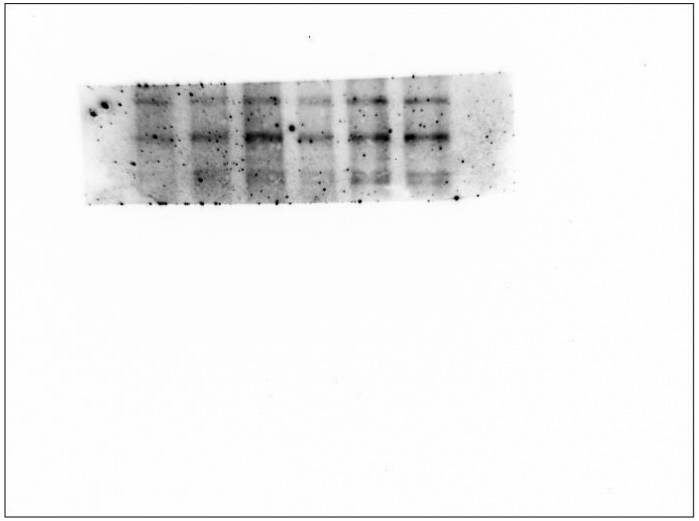

Chemiluminescence

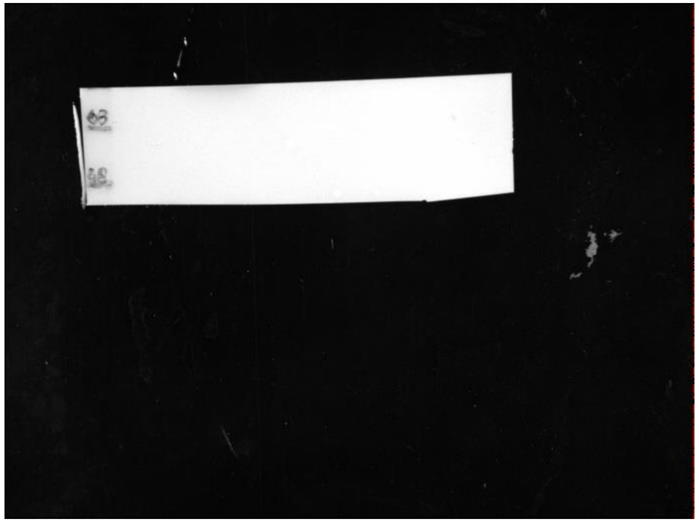

Original Blot

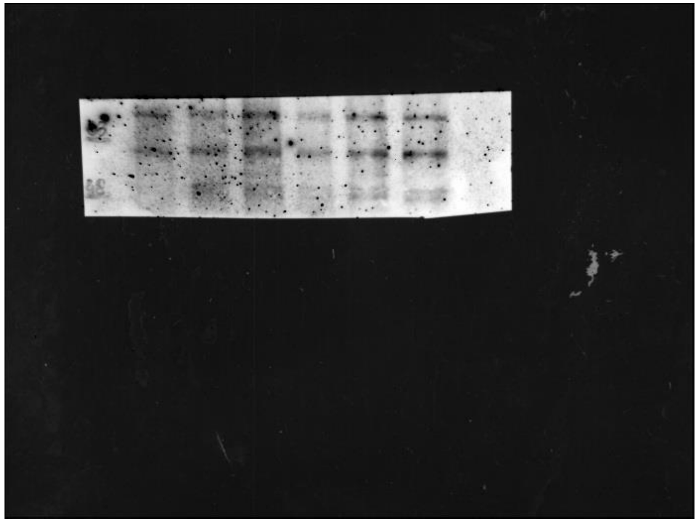

Merge

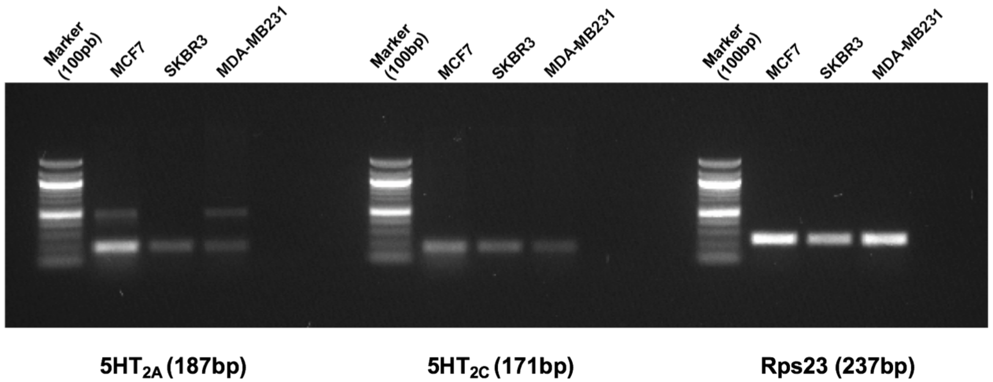
Marker (100pb)
MCF7
SKBR3
MDA-MB231
Marker (100bp)
MCF7
SKBR3
MDA-MB231
Marker (100bp)
MCF7
SKBR3
MDA-MB231
$5HT_{2A}$ (187bp)
$5HT_{2C}$ (171bp)
Rps23 (237bp)

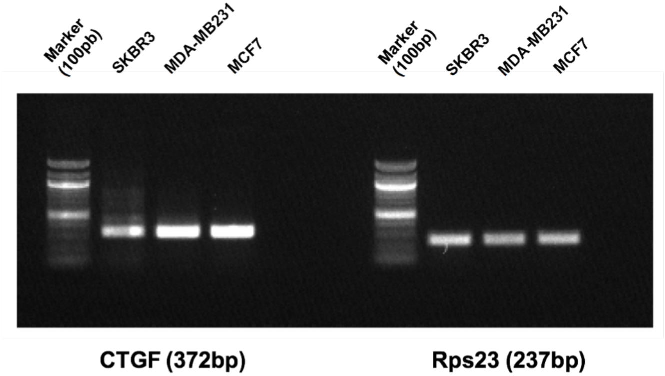
Marker (100pb)
SKBR3
MDA-MB231
MCF7
Marker (100bp)
SKBR3
MDA-MB231
MCF7
CTGF (372bp)
Rps23 (237bp)
